# Polychlorinated Biphenyl Exposures and Cognition in Older U.S. Adults: NHANES (1999–2002)

**DOI:** 10.1289/ehp.1306532

**Published:** 2013-11-25

**Authors:** Maryse F. Bouchard, Youssef Oulhote, Sharon K. Sagiv, Dave Saint-Amour, Jennifer Weuve

**Affiliations:** 1 Department of Environmental and Occupational Health, Université du Québec à Montréal Centre hospitalier universitaire (CHU) Sainte-Justine, Montréal, Québec, Canada; 2 Department of Environmental Health, Boston University School of Public Health, Boston, Massachusetts, USA; 3 Department of Psychology, Université du Québec à Montréal CHU Sainte-Justine, Montréal, Québec, Canada; 4 Rush University Medical Center, Chicago, Illinois, USA; 5 Department of Environmental Health, Harvard School of Public Health, Boston, Massachusetts, USA

## Abstract

Background: Polychlorinated biphenyls (PCBs) are ubiquitously present in humans because of their resistance to degradation and accumulation in fatty tissues. Data on neurotoxic effects in older adults are limited.

Objective: We examined the cross-sectional association between serum PCB concentrations and cognitive function in older adults from the general U.S. population.

Methods: We analyzed data from 708 respondents, 60–84 years of age, participating in the National Health and Nutrition Examination Survey (1999–2002). We used the summed concentrations of 12 lipid-standardized PCB congeners as the measure of exposure and assessed cognitive function with the Digit-Symbol Coding test. We adjusted analyses for age, education, race/ethnicity, and poverty/income ratio.

Results: The median concentration of lipid-standardized PCBs in serum was 271 ng/g (interquartile range, 193–399 ng/g). We found a significant interaction between dioxin-like PCB concentration and age in association with cognitive score (*p* = 0.04). Among older individuals (70–84 years of age), a 100-ng/g increase in serum concentrations of dioxin-like PCBs was associated with a significantly lower cognitive score (–2.7 points; 95% CI: –5.1, –0.2; *p* = 0.04); however, in younger individuals (60–69 years of age), there was a nonsignificant positive association (2.9 points; 95% CI: –1.8, 7.7; *p* = 0.32). Among the older participants, the negative association was more pronounced in women than in men.

Conclusion: Our findings support the hypothesis that PCB exposure has adverse cognitive effects even at levels generally considered to pose low or no risk, perhaps affecting mainly those of advanced age.

Citation: Bouchard MF, Oulhote Y, Sagiv SK, Saint-Amour D, Weuve J. 2014. Polychlorinated biphenyl exposures and cognition in older U.S. adults: NHANES (1999–2002). Environ Health Perspect 122:73–78; http://dx.doi.org/10.1289/ehp.1306532

## Introduction

Polychlorinated biphenyls (PCBs) were widely used in the United States from 1930 to 1979 in electric equipment and cooling systems and as plasticizers and solvents in many products, including adhesives, flame retardants, and carbonless copy paper (Agency for Toxic Substances and Disease Registry 2000). PCB production was banned in the United States in 1979, and the Stockholm Convention on Persistent Organic Pollutants banned PCB production and use in 2001. Despite being banned, concerns remain about the quality of disposal processes, which range from effective remediation to deliberate dumping, given that these contaminants resist degradation and persist in the environment. PCBs are lipophilic, bioaccumulate in marine life and animal tissues, and biomagnify in the food chain (Van Oostdam et al. 2005). As a result, PCBs are ubiquitously present in tissues of human populations.

Most research on the neurotoxic effects of PCB exposure has focused on childhood neurobehavioral impairments associated with prenatal exposure, with multiple studies reporting adverse associations between exposures and intelligence quotient and executive function as well as with processing speed, verbal abilities, visual recognition, and memory (as reviewed by Boucher et al. 2009). Fewer studies have addressed exposure to PCBs and neurobehavioral functions in adults, but these limited findings suggest that the effects of PCBs may vary by age and sex. A study of 50- to 90-year-old fish-eaters in the region of Lake Michigan reported a negative association between PCB levels in serum and cognitive function, particularly learning and memory (Schantz et al. 2001), but no association with executive and motor functions (Schantz et al. 1999). A study of 18- to 79-year-old Native American adults from the Saint Lawrence region in upstate New York reported an association between higher PCB exposures and poorer executive function, memory, and motor functions, but only among individuals > 40 years of age who had serum PCB levels above a threshold of 3 ppb (Haase et al. 2009). Among 55- to 74-year-old individuals living along contaminated portions of the upper Hudson River in New York, higher serum PCB levels were associated with poorer learning and memory among men, and with depressive symptoms among women (Fitzgerald et al. 2008).

A study of a highly exposed Taiwanese population ≥ 60 years of age with past exposures to PCBs and dibenzofurans through contaminated cooking oil reported an inverse association between blood PCB levels and performances on tests of attention, memory, and learning > 20 years after this high-exposure event, although only among women (Lin et al. 2008). A retrospective mortality study of 17,321 workers exposed occupationally from the 1940s through the 1970s reported significantly higher than expected mortality from amyotrophic lateral sclerosis, Parkinson disease, and noncerebrovascular dementia among women, although the numbers of deaths were very small (*n* = 10, 6, and 14, respectively) (Steenland et al. 2006).

Overall, findings on PCB exposure and cognition suggest sex- and age-related differences in patterns of association, with women and older adults being more susceptible. There are limited data on possible neurotoxic effects of PCB exposures in community-exposed older adults, despite the fact that exposure levels were higher in the past, resulting in older individuals having high PCB body burden relative to younger individuals (Nichols et al. 2007). In addition, the aging nervous system could be particularly vulnerable to neurotoxicant insults because of a lower compensation capacity (Weiss 2000). In the present study, we examined the cross-sectional association between serum PCB concentrations and cognitive function in a sample of older adults (60–84 years of age) representative of the noninstitutionalized general U.S. population.

## Methods

*Study design and population*. The National Health and Nutrition Examination Survey (NHANES) is a cross-sectional, population-based health survey of noninstitutionalized U.S. residents conducted by the National Center for Health Statistics (NCHS) of the Centers for Disease Control and Prevention (CDC). NHANES uses a complex, multistage probability sampling design, with oversampling of certain subgroups. Participants completed household surveys that included questions about demographics and health history, and they provided blood and urine samples collected during physical examinations at mobile centers. The study protocol has been described previously (CDC 2012). NHANES was approved by the NCHS Institutional Review Board, and all participants provided written informed consent. We merged data from the survey years 1999–2000 and 2001–2002. Cognitive function was assessed only in respondents ≥ 60 years of age, and age is top-coded at 85 years in NHANES data releases for confidentiality reasons. Therefore, our study population included individuals 60–84 years of age.

*Measurements of PCBs in serum*. Serum PCB congeners were measured on a randomly selected subsample that included one-third of NHANES participants. Analytes were measured by high-resolution gas chromatography/isotope-dilution high-resolution mass spectrometry (HRGS/ID-HRMS). NHANES provides congener concentrations both on a weight–weight basis and lipid adjusted; we used the latter in our analyses.

Nine dioxin-like and 14 non–dioxin-like PCB congeners were measured in serum samples, but only the 12 congeners detected in > 75% of individuals were retained in our analyses. Three PCB exposure metrics were calculated to reflect alternative assumptions relating to neurotoxicity (Faroon et al. 2001): *a*) the sum of 5 dioxin-like PCBs (congeners 74, 118, 156, 126, and 169); *b*) the sum of 7 non–dioxin-like PCBs (congeners 99, 138, 146, 153, 170, 180, and 187); and *c*) the sum of all 12 PCB congeners.

In a complementary analysis, we applied toxic equivalency factors published by the World Health Organization (WHO) in 2005 to account for the relative toxicity and concentration of dioxin-like congeners 118, 156, 126, and 169 (van den Berg et al. 2006), and we summed the values to obtain the toxic equivalents.

For PCB measures at or below the detection limit, we imputed values using regression on order statistics (Helsel 2005), rather than using the common approach of imputing these values as the detection limit divided by the square root of 2. Regression on order statistics assumes an underlying log-normal distribution and uses quantile regression to predict PCB concentrations from those observed at or below the detection limit. This is a robust and less-biased procedure with a smaller variance than other methods under the log-normal distribution (Shumway et al. 2002). For some individuals with missing data for a few congeners only (between 0% and 11% of observations, depending on the congener), we imputed values using chained equations (van Buurren et al. 1999), as described in greater detail below.

*Assessment of cognitive function*. The Wechsler Adult Intelligence Scale (Third Edition) Digit-Symbol Coding Test (Wechsler 1997) was administered to participants ≥ 60 years of age during the NHANES household interview (CDC 2005). The task consisted of drawing symbols under the corresponding number, using the key provided at the top of the exercise form. The score was the number of correct symbols drawn within 2 min.

The Digit-Symbol Coding Test is used to detect brain impairment in clinical settings. It primarily evaluates processing speed and motor ability, but recent evidence suggests that it may also reflect memory and executive function (Joy et al. 2004; Wang et al. 2011). Because these functions often diminish with advancing age, this test also is used in studies of cognitive aging. This was the only test of cognitive assessment in the NHANES protocol.

*Statistical analysis*. Of the 3,297 participants in the age range of 60–84 years, 870 were included in the random subsample with serum PCB measurements. Of these, we excluded 5 individuals with data for only one dioxin-like congener. A cognitive score was available for 760 of the remaining 865 participants; cognitive scores were missing for various reasons such as the test was not administered because there was too much distraction (*n* = 42), refusal (*n* = 10), unable to complete the test for physical (*n* = 12) or cognitive (*n* = 9) limitation, or other reasons (*n* = 32). We excluded 52 individuals who reported a history of stroke, leaving 708 individuals in the analytical data set. Cognitive scores were normally distributed (data not shown).

Associations between lipid-standardized serum PCB concentrations or toxic equivalents and cognitive performance were estimated using general linear models. Given that dioxin-like and non–dioxin-like congeners might have different relations to cognition (Faroon et al. 2001), we examined them separately. In these models, we used serum PCB concentrations as continuous values. Toxic equivalents were log-transformed (base 10) to address skewness. We used the Survey package in R (http://www.r-project.org/) to obtain estimates of association and confidence intervals (CIs) accounting for the multistage probability sampling design of NHANES (Lumley 2012). We also used the weights to adjust for the oversampling of certain population subgroups and to account for nonresponse and noncoverage in NHANES. All tests were two sided, and *p* < 0.05 was the level of significance.

Previous studies reported age- and sex-related differences in the association of PCB exposures with neuropsychological function (Fitzgerald et al. 2008; Haase et al. 2009). To explore these differences, we included cross-product terms in our models. We ran models including two-way (age × PCB and sex × PCB) and three-way interaction terms (sex × age × PCB) in addition to lower-order terms. Age and PCB concentrations were modeled as continuous variables in these initial analyses to determine whether significant interactions were present. For further analyses, we divided age into two groups (split at the median, 69 years) in order to perform age-stratified analyses. To account for residual confounding by age within age strata, we also included a term for age with continuous values in years. To make the resulting associations easier to interpret, we plotted the results from similar age- and sex-stratified analyses in which we grouped serum PCB concentrations into tertiles.

Models were adjusted for likely sources of confounding based on previous literature and associations with serum PCB concentration. These variables were age (years, continuous), education (less than 9th grade, 9th–11th grade, high school graduate, some college, college graduate and above), poverty/income ratio [PIR (the ratio of self-reported family income to the family’s appropriate threshold value), grouped into quartiles], and race/ethnicity (non-Hispanic white, non-Hispanic black, Mexican American, multiracial or other). Additional adjustment for age squared, blood lead levels, alcohol consumption, and smoking changed the association estimates by < 10% (data not shown), so we did not include these variables in the final models.

Some participants were missing data on PCB congeners (at most, 4.5% were missing data for a given congener), PIR (*n* = 79), and education (*n* = 1). We imputed these values using multiple imputations by chained equations (MICE), an approach that uses all the variables used in the models to impute the missing values (van Buuren et al. 1999). We conducted a sensitivity analysis excluding participants with missing values for education and PIR. Another sensitivity analysis consisted of excluding individuals with very low cognitive scores (i.e., < 5th percentile). Finally, we introduced a separate term for lipids into the models instead of using lipid-standardized concentrations as a sensitivity analysis.

## Results

*Descriptive statistics*. Among the participants in our study, the median lipid-standardized serum concentrations were 49, 223, and 271 ng/g for dioxin-like, non–dioxin-like, and total PCBs, respectively (Table 1). Serum concentrations of all individual PCB congeners were significantly correlated with total PCB concentration (data not shown), and PCB 153 had the highest correlation (Pearson *r* of logged values = 0.98). We also found a strong correlation between dioxin- and non–dioxin-like PCB concentrations (Pearson *r* of logged values = 0.81 for lipid-standardized concentrations). In weighted univariate analyses, total lipid-standardized serum PCB concentrations did not vary by sex, education, PIR, or tobacco use (all *p* > 0.20), but concentrations did vary significantly by race/ethnicity and were higher in older participants (*p* < 0.001). Non-Hispanic black participants had higher concentrations than non-Hispanic white participants (*p* < 0.001), and Mexican-American participants and other groups had lower levels than non-Hispanic white participants (*p* < 0.05). Table 2 presents unweighted statistics on mean concentration of total serum PCBs with respect to population characteristics.

**Table 1:** Distribution of serum PCB concentrations in the study population (statistics not weighted; *n* = 708), NHANES 1999–2002, individuals 60–84 years of age.

PCB metric	Minimum	Percentile					Maximum
		5	25	50	75	95	
Non-lipid adjusted [wet weight (ng/g)]
Dioxin-like PCBs	0.06	0.10	0.20	0.33	0.50	1.03	3.78
Non–dioxin like PCBs	0.28	0.51	1.01	1.45	2.13	3.90	17.10
Total PCBs	0.35	0.66	1.25	1.79	2.57	4.99	20.88
Lipid-standardized (ng/g)
Dioxin like PCBs	8	16	31	49	76	151	507
Non–dioxin like PCBs	36	80	157	223	330	569	1,286
Total PCBs	43	102	193	271	399	710	1,513

**Table 2:** Concentration of total serum PCBs (wet weight and lipid-standardized) by population characteristics (statistics not weighted; *n* = 708), NHANES 1999–2002, individuals 60–84 years of age.

Characteristic	*n* (%)	Wet weight PCBs (ng/g) GM (GSD)	Lipid-standardized PCBs (ng/g lipid) GM (GSD)
Total population	708 (100)	1.81 (1.82)	275 (1.78)
Sex
Male	334 (47)	1.73 (1.85)	273 (1.78)
Female	374 (53)	1.89 (1.80)	277 (1.78)
Age (years)
60–64	201 (28)	1.63 (1.80)	241 (1.78)
65–69	206 (29)	1.75 (1.71)	262 (1.68)
70–74	132 (19)	1.88 (1.91)	295 (1.86)
75–79	94 (13)	1.87 (1.85)	289 (1.74)
80–84	75 (11)	2.38 (1.86)	370 (1.74)
Race/ethnicity
Non-Hispanic white	419 (59)	1.86 (1.75)	283 (1.67)
Non-Hispanic black	99 (14)	2.54 (1.77)	410 (1.74)
Mexican American	144 (20)	1.42 (1.78)	206 (1.76)
Multiracial/other	46 (7)	1.47 (1.97)	217 (1.86)
Education
< 9th grade	162 (23)	1.51 (2.07)	225 (2.05)
9th–11th grade	117 (17)	1.98 (1.75)	303 (1.72)
High school graduate	169 (24)	1.90 (1.68)	288 (1.65)
Some college	145 (21)	1.91 (1.68)	288 (1.66)
College graduate	114 (16)	1.86 (1.84)	290 (1.68)
Missing	1 (0.1)	— (—)	— (—)
PIR quartiles
1 (≤ 1.21)	165 (23)	1.67 (1.88)	244 (1.80)
2 (1.22–2.15)	143 (20)	1.71 (1.80)	264 (1.74)
3 (2.16–3.49)	160 (23)	1.92 (1.85)	290 (1.83)
4 (≥ 3.50)	161 (23)	1.89 (1.76)	294 (1.72)
Missing	79 (11)	1.95 (1.80)	288 (1.74)
Tobacco use
Smoker	93 (44)	1.73 (1.86)	264 (1.86)
Ever-smoker	307 (13)	1.77 (1.79)	267 (1.76)
Never-smoker	308 (43)	1.89 (1.83)	287 (1.77)
Abbreviations: GM, geometric mean; GSD, geometric standard deviation.			

Performance on the cognitive test ranged widely among the study participants, with scores ranging from 0 to 113 points (mean ± SD, 43.1 ± 18.4). Scores decreased significantly with age (*p* < 0.001), and there was no significant difference in scores between women and men after taking age into account (means of 44.2 and 42.0, respectively; *p* = 0.1). Also after taking age into account, scores were significantly lower among participants who had less formal education and lower PIRs (*p* < 0.001), but there was no difference with respect to tobacco use (*p* = 0.84). There was no significant difference in the mean PCB concentration between individuals with and without a cognitive score available (*p* > 0.4 for dioxin-like, non–dioxin-like, and total PCBs).

*Association of serum PCB concentration with cognitive score*. The total serum PCB concentration was not significantly associated with cognitive score in analyses adjusted for age, sex, education, race/ethnicity, and PIR (a 100-ng/g increment in lipid-standardized total PCB concentration was associated with a 0.16-unit lower cognitive score; 95% CI: –0.87, 0.55; *p* = 0.6). Likewise, dioxin-like and non–dioxin-like PCB concentrations were not significantly associated with cognitive score (differences in cognitive scores of –1.05 points; 95% CI: –3.79, 1.70; *p* = 0.4 and –0.16 points; 95% CI: –1.03, 0.71; *p* = 0.7, respectively). For comparison, a 1-year increase in age was associated with a 0.87-point lower score (95% CI: –1.06, –0.68, *p* < 0.001) after adjusting for sex, education, race/ethnicity, and PIR.

We found no significant interaction between serum concentration of PCBs and sex in relation to cognitive score (data not shown). However, we observed a significant interaction between age and serum concentration of dioxin-like PCB congeners (*p* = 0.04). Among the older group (70–84 years of age), a 100-ng/g increase in dioxin-like serum PCBs concentration was associated with a 2.65-point lower cognitive score (95% CI: –5.13, –0.16; *p* = 0.04) (Table 3). By contrast, the association was positive though not significant for the younger group (60–69 years of age, difference in cognitive score = 2.92 points, 95% CI: –1.81, 7.66; *p* = 0.22). For total PCBs and non–dioxin-like PCBs, interactions with age were not significant (interaction *p*-values of 0.25 and 0.37, respectively) and there was no indication of associations of either exposure with cognitive scores in either age group (Table 3).

**Table 3:** Age-stratified, multivariable-adjusted differences in cognitive score (95% CI) per 100-ng/g increase in lipid-standardized serum PCB concentrations (*n* = 708), NHANES 1999–2002, individuals 60–84 years of age.

PCB metric	60–69 years of age (*n *= 361)		70–84 years of age (*n *= 347)	
	Difference in cognitive score^*a*^ (95% CI)	*p*-Value	Difference in cognitive score^*a*^ (95% CI)	*p*-Value
Dioxin-like PCBs	2.92 (–1.81, 7.66)	0.22	–2.65 (–5.13, –0.16)	0.04
Non–dioxin-like-PCBs	–0.06 (–1.41, 1.29)	0.93	–0.14 (–1.14, 0.87)	0.78
Total PCBs	0.06 (–1.09, 1.21)	0.91	–0.25 (–1.04, 0.55)	0.53
Association estimates were obtained with general linear models using sampling weights and accounting for the complex survey design. Missing data on PCB congeners, PIR, and education were imputed with MICE. PCB measurements below detection limit were imputed with regression on order statistics. ^***a***^Adjusted for age, sex, race/ethnicity, education, and PIR.				

**Figure 1:**
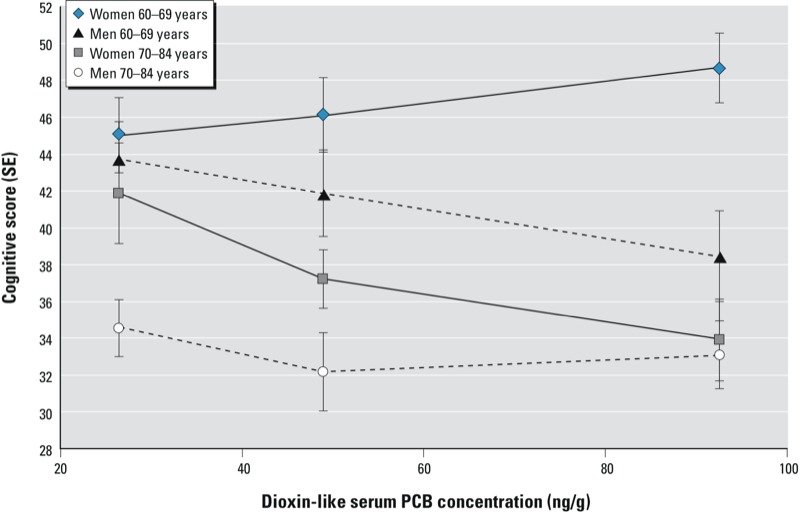
Mean cognitive score (SE) per tertile of lipid-standardized dioxin-like PCB concentration in serum (ng/g lipid) for men and women of 60–69 and 70–84 years of age, adjusted for age (years, continuous), race/ethnicity, education, and PIR (*n *= 708). Median concentrations for dioxin-like PCB tertile 1, 26 ng/g (range, 8–37); for tertile 2, 49 ng/g (37.1–63); and for tertile 3, 93 ng/g (63.1–507). Estimates obtained with general linear models using sampling weights and accounting for the complex survey design.

Further analysis showed that the 3-way interaction term, sex × age × PCB, was significant for dioxin-like PCB congeners concentration (*p* = 0.049), but not for total PCB concentration (*p* = 0.12) and non–dioxin-like PCB concentration (*p* = 0.16). Figure 1 shows mean cognitive scores by dioxin-like serum PCB concentration tertiles in age- and sex-stratified analyses. Among individuals 60–69 years of age, higher exposure was associated with lower cognitive scores in men and higher scores in women. Table 4 shows that, for women in the younger group, those in tertile 3 had scores 3.64 points higher than those in tertile 1 (95% CI: –1.67, 8.95; *p* = 0.13). For men in the younger group, those in tertile 3 had scores 5.29 points lower than men in tertile 1 (95% CI: –11.29, 0.71; *p* = 0.10). Among older individuals (70–84 years of age), we observed lower scores with higher exposure level for both men and women. For men in the older age group, those in tertile 3 had scores 2.37 points lower than those in tertile 1 (95% CI: –7.24, 2.49; *p* = 0.48). Of all groups, women in the older age group had the largest score difference between exposure levels, with those in tertile 3 having scores 7.94 points lower than those in tertile 1 (95% CI: –15.12, –0.76; *p* = 0.06). Supplemental Material, Table S1, presents serum PCB concentrations stratified for both sex and age.

**Table 4:** Age-stratified, multivariable-adjusted differences in cognitive score (95% CI) between lipid-standardized dioxin-like PCB tertiles (*n* = 708), NHANES 1999–2002, individuals 60–84 years of age.

Age group	Sex	Exposure levels comparison	Cognitive score difference (95% CI)	*p*-Value
60–69 [years (*n *= 361)]	Women	T2 (*n *= 78) vs. T1 (*n *= 63)	1.08 (–4.66, 6.83)	0.68
		T3 (*n *= 68) vs. T1 (*n *= 63)	3.64 (–1.67, 8.95)	0.13
	Men	T2 (*n *= 48) vs. T1 (*n *= 77)	–1.87 (–6.88, 3.14)	0.45
		T3 (*n *= 27) vs. T1 (*n *= 77)	–5.29 (–11.29, 0.71)	0.10
70–84 [years (*n *= 347)]	Women	T2 (*n *= 51) vs. T1 (*n *= 24)	–4.65 (–10.54, 1.24)	0.20
		T3 (*n *= 90) vs. T1 (*n *= 24)	–7.94 (–15.12, –0.76)	0.06
	Men	T2 (*n *= 58) vs. T1 (*n *= 72)	–2.37 (–7.24, 2.49)	0.22
		T3 (*n *= 52) vs. T1 (*n *= 72)	–1.46 (–7.07, 4.15)	0.48
T, tertile. Estimates were obtained with general linear models using sampling weights and accounting for the complex survey design. We used subpopulation analyses for age groups (< 70/≥ 70 years), and included a sex × tertile of PCBs inter­action term in the models to obtain sex- and age-specific cognitive score differences between tertiles of PCBs. Missing data on PCB congeners, PIR, and education were imputed with MICE. PCB measurements below detection limit were imputed with regression on order statistics.				

We observed a significant interaction between age and toxicity equivalents (*p* = 0.003) in association with cognitive scores. A 10-fold increase in toxic equivalents was associated with a 5.74-point lower cognitive score (95% CI: –9.48, –2.00; *p* = 0.01) among the older age group, compared with a nonsignificant 2.80-point higher score in the younger age group (95% CI: –2.80, 8.40; *p* = 0.34). In addition, the 3-way interaction term, sex × age × toxicity equivalents approached significance (*p* = 0.06). Among men, a 10-fold increase in toxic equivalents was associated with a 2.89-point lower cognitive score (95% CI: –11.63, 5.86; *p* = 0.53) in the younger age group and a 5.15-point lower cognitive score (95% CI: –12.64, 2.35; *p* = 0.20) in the older age group. In women, we observed significantly lower cognitive score with higher toxic equivalents in the older age group (–9.06 points, 95% CI: –14.54, –3.58; *p* = 0.005). The association was positive but not significant in younger women (5.46 points, 95% CI: –1.44, 12.35; *p* = 0.15).

We conducted sensitivity analyses excluding individuals with missing PIR and education values, as well as individuals with very low cognitive scores, and these yielded results similar to those from our primary analyses. Finally, introducing a separate term for lipids into the models instead of using lipid-standardized concentrations did not change the results.

## Discussion

In this study of older adults (60–84 years of age), higher exposures to PCBs, as indicated by serum concentrations of the sum of five dioxin-like PCB congeners, were associated with lower cognitive function scores among the oldest participants (70–84 years of age). Whereas all three PCB exposure measures (total, dioxin-like and non–dioxin-like congeners) were negatively associated with cognitive scores in this age group, only the association with dioxin-like PCBs was clearly different from the null. These findings support previously reported associations of poorer cognitive performance in cohorts of individuals with higher PCB exposure levels (Fitzgerald et al. 2008; Haase et al. 2009; Lin et al. 2008; Schantz et al. 2001). Our findings extend these observations to the general U.S. population of older adults. Aging persons could be at risk because of higher cumulative exposure built up across a lifetime, susceptibility due to underlying medical conditions (e.g., vascular disorders), and diminished cognitive reserve capacity. The association between PCBs and cognitive scores was strongest among older women (70–84 years of age), for whom the difference in cognitive scores associated with the highest versus tertile 1 of dioxin-like PCBs (–7.9 points) was comparable to the difference in cognitive scores between participants in our study who were 8.8 years apart in age.

The stronger association seen in older women supports evidence from another epidemiological study that reported poorer scores on tests of attention, memory, and learning with higher blood PCB levels only in females (Lin et al. 2008). Furthermore, a study based on brain imaging showed that lower dopamine transporter density was associated with higher serum PCB levels among women (*n* = 39), but not men (*n* = 50), who worked as capacitor workers (Seegal et al. 2010). In addition, perinatal exposure to PCBs was associated with spatial learning deficits lasting until adult age in female rats but not in male rats (Schantz et al. 1995). Sex-specific associations between PCBs and cognitive function might be explained by estrogenic effects of these compounds, which have been demonstrated *in vitro* (DeCastro et al. 2006). A large body of literature suggests that sex hormones, primarily estrogen, may influence neurobehavioral performances (Petrovska et al. 2012), although the relationships are complex. In contrast, among the younger women in our study, those with higher levels of exposure performed better on the cognitive test. We do not know of a biological mechanism that would explain this finding.

The present study has limitations, notably its cross-sectional design. It is possible that confounding accounts, at least in part, for the associations we observed. For example, a common source of PCB exposure is consumption of contaminated fish (Turyk et al. 2007). Fish can also contain methylmercury, a neurotoxicant, and omega-3 fatty acids, which may be neuroprotective (Turyk et al. 2012). Accounting for these factors in analyses of PCB exposures and cognition could yield estimates of association that are smaller or larger. Unfortunately, data on mercury exposure and omega-3 fatty acid intake were not available for our study participants (mercury was only measured in NHANES female participants between 16 and 49 years of age). By adjusting our analyses for several other important potential sources of confounding, such as age and socioeconomic status (education and income), we may have partially accounted for these other exposures. Nonetheless, future research should evaluate the combined cognitive effects of PCB exposures, mercury exposure, and omega-3 fatty acids. Finally, there might be a cohort effect, with higher PCB levels in older participants reflecting a population-level cohort effect related to the timing of peak exposures experienced by the older individuals before PCB production and use was regulated or banned. Therefore, our finding might not apply to newer cohorts of old individuals who did not experience such high levels of exposure.

A strength of the present study is its large sample size (to our knowledge, the largest to date), which allowed for age- and sex-stratified analyses. The negative association between serum PCBs and cognitive scores in the older participants [i.e., those ≥ 70 years of age, but not younger participants (60–69 years of age)] might reflect a diminished reserve capacity in the older participants. The nervous system has a great capacity to compensate for toxic insult, but this capacity could be reduced with aging (Grandjean 1991). Recent research findings of functional imaging investigations have suggested that certain neuromotor and cognitive deficits observed in normal aging may be mediated by changes in the striatal dopamine system (Backman et al. 2000). Experimental data strongly suggest that PCBs disrupt the activity of the dopaminergic system (Faroon et al. 2001); the negative association between PCBs and cognitive scores among older individuals could, therefore, reflect changes in this neurotransmitter system. Furthermore, exposure to PCBs was associated with an increased risk of type 2 diabetes among Nurses’ Health Study participants (Wu et al. 2012), and type 2 diabetes is itself a strong risk factor for cognitive impairment and dementia (Strachan et al. 2011). Finally, PCBs were associated with hypertension in NHANES participants (based on a population that overlapped with the present study), which suggests that negative associations with cognitive scores might also reflect vascular effects (Everett et al. 2008).

Associations appeared to be limited to the dioxin-like PCBs, including the WHO toxic equivalents for dioxin-like congeners. This latter finding is consistent with, although certainly not exclusive to, effects on cognition via activation of the aryl hydrocarbon receptor. Emerging animal and *in vitro* data support this possibility (Latchney et al. 2013; Williamson et al. 2005; Xie et al. 2013); however, more research is needed to confirm this potential mechanism.

## Conclusion

Our findings suggest that PCB neurotoxicity may contribute to cognitive deficits in older persons, even at levels generally considered to pose low or no risk. These findings indicate that PCBs still deserve consideration for their impact on the health of the population. Indeed, although PCBs are now banned in the United States, the decline of serum concentrations in the population has been slow for persons ≥ 40 years of age, reflecting the persistent nature of these contaminants (LaKind et al. 2009). Thus, it is likely that, even though PCBs have been banned, their legacy will continue in living populations.

## Supplemental Material

(229 KB) PDF
