# Supplementary material for: Polychlorinated Biphenyl Exposures and Cognition in Older U.S. Adults: NHANES (1999–2002)
Source: Environ Health Perspect. 2013 Nov 25;122(1):73–8. doi: 10.1289/ehp.1306532 (PMC3888566; doi:10.1289/ehp.1306532)
Supplement: (229 KB) PDF [file ehp.1306532.s001.508.pdf]

Environmental Health Perspectives doi:1306532

Supplemental Material

Polychlorinated Biphenyl Exposures and Cognition in Older U.S. Adults: NHANES (1999–2002)

Maryse F. Bouchard, Youssef Oulhote, Sharon K. Sagiv, Dave Saint-Amour, and Jennifer Weuve

Table S1. Distribution of serum PCB concentrations in the study population, stratified for sex and age (n = 708). Statistics not weighted, NHANES 1999-2002, individuals 60 to 84 years.

		Percentiles					Percentiles			
	min	25	50	75	max	min	25	50	75	max
**Women (n = 374)[a]**										
Not lipid-standardized (wet weight), ng/g										
Dioxin-like PCBs	0.06	0.23	0.36	0.49	1.93	0.06	0.31	0.45	0.71	2.08
Non-dioxin-like PCBs	0.30	0.91	1.36	1.94	7.26	0.37	1.17	1.68	2.56	9.77
Total PCBs	0.40	1.16	1.74	2.40	8.28	0.44	1.50	2.12	3.41	11.82
Lipid-standardized, ng/g										
Dioxin-like PCBs	10	34	50	71	245	9	47	70	102	366
Non-dioxin-like PCBs	40	132	194	279	1205	57	166	252	379	1285
Total PCBs	52	171	246	362	1374	69	216	320	475	1446
**Men (n = 334)[b]**										
Not lipid-standardized (wet weight), ng/g										
Dioxin-like PCBs	0.06	0.16	0.23	0.36	1.19	0.06	0.18	0.27	0.43	3.78
Non-dioxin-like PCBs	0.28	1.00	1.33	2.04	7.63	0.31	1.07	1.48	2.12	17.10
Total PCBs	0.35	1.20	1.60	2.38	8.04	0.37	1.28	1.76	2.58	20.88
Lipid-standardized, ng/g										
Dioxin-like PCBs	8	24	36	51	197	10	29	44	70	506
Non-dioxin-like PCBs	36	157	220	287	1134	48	176	246	343	1164
Total PCBs	44	181	256	336	1217	58	216	296	421	1513

[a]Age 60-69 years (n = 209); age 70-84 years (n = 165).

[b]Age 60-69 years (n = 152); age 70-84 years (n = 182).
